# Impact of Physical Exercise on Symptoms of Depression and Anxiety in Pre-adolescents: A Pilot Randomized Trial

**DOI:** 10.3389/fpsyg.2019.01820

**Published:** 2019-08-08

**Authors:** Arnaud Philippot, Alexandre Meerschaut, Laura Danneaux, Gauthier Smal, Yannick Bleyenheuft, Anne G. De Volder

**Affiliations:** ^1^MSL-In Laboratory, Institute of Neuroscience, Université catholique de Louvain, Brussels, Belgium; ^2^Psychiatric Hospital, AREA+, Epsylon ASBL, Brussels, Belgium; ^3^Pediatric Neurology Service, Cliniques Universitaires Saint-Luc, Brussels, Belgium

**Keywords:** exercise medicine, depression, anxiety, youth, students, move and feel good clinical trial

## Abstract

**Aim:**

The intensity of the most appropriate exercise to use in depressed youth is unclear due to differences in methodology and the lack of evidence documenting the effect of physical activity in children. Therefore, the authors of this study attempted to document the effectiveness of different training intensities to reduce symptoms of depression and anxiety in pre-teens.

**Methods:**

The study included twenty-seven, randomly selected pre-adolescents (aged between 9–11 years of age) all of whom had Primary education. The participants were enrolled and, over a 5-week period, were subject to either intensive or low-to-moderate exercise programs four times a week. Psychological self-reports, as well as physical examinations, were conducted before and after such programs in blinded assessments. Psychological effects were considered the primary outcome, whilst physical condition was secondary.

**Results:**

Four subjects were lost and twenty-three were analyzed. General linear model with 2 criteria revealed significant changes (*p* = 0.05) in trait anxiety symptoms over time in the low-to-moderate intensity group (LMIG). Within group changes followed a significant decrease in levels of anxiety (38.82 ± 2.20 to 33.36 ± 2.83, *p* = 0.004) and depression (10.36 ± 2.83 to 6.73 ± 1.88, *p* = 0.006) related symptoms amongst those in the LMIG.

**Interpretation:**

This study indicated that depression and anxiety symptoms were reduced amongst a non-clinical sample of Primary educated pre-adolescents when they were subject to a low-to-moderate exercise program. The program focused on associating movement with pleasure, encouraged positive and non-competitive interactions between participants.

**Clinical Trial Registration:**

www.ClinicalTrials.gov, identifier NCT02970825, autumn 2016, updated May 7, 2018 (https://clinicaltrials.gov/ct2/show/NCT02970825).

## Introduction

Although often clinically undiagnosed, symptoms of depression and anxiety can be seen in pre-adolescent children aged 9 to 12. These signs often go unrecognized, despite their prevalence: 8–9% of children and adolescents show depressive symptoms (Mehler-Wex and Kolch, 2008) and levels of anxiety disorders range from 2.5–30% due to the heterogeneity of diagnostic methods (Salum et al., 2013). Detecting these symptoms at an early stage is important in the treatment of present and future mental-health issues; children and pre-adolescents with early signs of depression are more likely to have severe depression later in life (Korczak et al., 2017b). Nonetheless, the optimal treatment for those symptoms in this age bracket should focus on non-pharmaceutical options, as anti-depressant medication is shown to increase the risk of suicidal thoughts amongst children (Hammad et al., 2006).

There are many cohort studies or Randomized Controlled Trials (RCT) indicating that physical activity (PA) or adapted physical exercise programs are associated with decreased depressive symptoms in adulthood. There is also evidence that indicates that physical exercise reduces levels of depression in adolescents and pre-adolescents. However, additional studies with higher methodological quality are needed to corroborate these findings (Carter et al., 2016; Korczak et al., 2017a; Radovic et al., 2017).

An important question is “what is the most appropriate exercise intensity in children and adolescents?” The few studies in this issue have yielded contradictory results. The first study showed no therapeutic difference as per the intensity of the exercise for children and young people up to the age of 20 (Larun et al., 2006). A program of varying intensity, based on individual preferences, reduced symptoms of depression in a sample of 87 adolescents (Carter et al., 2015). According to a meta-analysis, a low or moderate intensity exercise program (three times a week for 6 to 12 weeks) is most effective in reducing depression scores in this age group (Carter et al., 2016). Another systematic review found that more frequent and intense sessions were associated with fewer depressive symptoms (Korczak et al., 2017a). The limited amount of meta-analyses available incurs a high level of inter-study heterogeneity, in terms of intervention methods and outcome measures. This is especially apparent in children and adolescents in non-clinical settings (Larun et al., 2006; Carter et al., 2016). To date, there is no evidence that indicates PA or adapted exercise programs have a negative effect on psychological status.

Researchers have attempted to evaluate the link between fit physical condition and depression. One meta-analysis clearly indicated that there is a negative correlation between fit behavior in young people and depressive behavior (Korczak et al., 2017a). A previous cohort found a possible buffering effect of vigorous exercise on the relationship between stressful life events and depression in a sample of adolescents (12–15 years), whereby a low level of PA and high levels of sedentary lifestyle predicted a high score of depressive symptoms (Sund et al., 2011). In addition, a RCT with a high methodological quality [confirmed 6/10 score according to the Physiotherapist Evidence Database (PEDro scale)] showed that adolescents meeting the DSM-IV MDD criteria reduced their score of depression from 6 weeks of intervention (3 sessions per week) with vigorous intensity [>12 Kilocalories/Kilogram/Week (KKW)] compared to low intensity (<4 KKW) exercise (Hughes et al., 2013). Another point of view is that these behaviors seemed to be closely linked with the cognitive functions. At present, they found some evidence that hippocampal volume could be bigger in more fit compared to less fit pre-adolescents. A bigger hippocampal volume would be related to better memory tasks (Chaddock et al., 2010). Then cognitive function scored lower in children and adolescents with depressive disorder (Wagner et al., 2015). The cingulate cortex and prefrontal areas seem to exhibit altered functioning in depressive patients and this appeared to explain the poor performance in cognitive processes (Belzung et al., 2015). Eventually, considering that better cardiovasuclar fitness may enable improving the cognitive function and seems to protect from depressive symptoms and we provide a short time period of intervention (5 weeks), we could support the notion that a high-intensity program may be effective in reducing depressive symptoms in children. In fact, the purpose of this intensity would be to improve the participants’ physical condition by giving a protect effect on the mental health compared to a lower intensity that could change the physical condition to a lesser extent in a 5 weeks duration of experiment.

The main objective of this study was to investigate the impact of structured PA on the prevention of depressive and anxiety symptoms in a non-clinical sample of schoolchildren. A low- to moderate-intensity PA was compared to a high-intensity PA. We also sought to evaluate whether such physical activities, implemented in the form of games, could affect the above-mentioned symptoms. We hypothesized that high-intensity PA would lead to greater psychological improvement than low- to moderate-intensity activity, assuming that physical condition was closely linked to individual psychology.

Anxiety and depressive symptoms have common risk factors, indicating that a similar prevention for both disorders may be useful in many cases. Therefore, we studied the common effect of PA on these symptoms (Belhadj Kouider and Petermann, 2015).

## Methods

### Participants

Participants were recruited from a primary school on a voluntary basis and the study was presented in classrooms. The inclusion criteria were as follows: (1) Be involved in an official education program; (2) aged between 9 and 12 years; (3) acceptance of the randomization principle; (4) absence of neurological or psychiatric history; (5) absence of uncorrected sensory disorder preventing the understanding of instructions; and (6) absence of conduct disorders. The exclusion criteria were as follows: (1) refusal to participate; (2) Body Mass Index above P95; (3) unstable diabetes; (4) coagulation disorders (apart from specific exceptions and medical authorization); (5) severe and unstable asthma; (6) history of heart malformation or cardiovascular disease; and (7) medical conditions prohibiting sport or PA. All parents completed a medical questionnaire before starting the research. Accordingly, only the children who met the inclusion and exclusion criteria were included. The others (who were treated by medications or therapy) were kindly excluded.

The study was undertaken at the “elementary school Parmentier – Jean XXIII” in Brussels and its facilities (a classroom, gymnasium, and park). Participants were informed that they would not be allowed to choose the group to which they would be assigned and could leave the study at any time if they wished. Their parents provided their written informed consent before the start of the experiment. The ethical Committee of the School of Medicine of the Université catholique de Louvain approved the study.

### Design

We conducted a low- to moderate-intensity intervention and a high-intensity intervention in parallel, focusing on pleasure, interaction and non-competition. Each intervention took place for 5 weeks at a rate of four sessions per week (Monday, Tuesday, Thursday, and Friday) during the lunch hour. Thus, the study followed children from two groups for a total of 20 sessions that lasted 50 min. There was an assessment period in the week before and the week after the program, where tests of the participants were gathered. Quantifiers for the randomization included individual scores for the CDI, BMI, duration of the Léger shuttle test, age, sex, and school year. Thereafter, the principal investigator assorted the participants into two equivalent groups. Randomization at the group level was done by an external blind researcher who provided the intervention allowance by e-mail. Eventually the participants who met the inclusion criteria were assigned randomly to either the HIG (*n* = 14) or the LMIG (*n* = 13) based on the pre-evaluation. During the intervention sessions, therapists and participants were not aware of individual scores used for randomization. The two pairs of therapists (two physical educators and two physiotherapists) managed the same number of sessions in each group alternately. The principal investigator provided medical security as well as the components of each program.

The Metabolic Equivalent of Task (MET) was used to define the intensity of interventions (Ainsworth et al., 2000). The World Health Organization (WHO) saw that an activity included between 1.6 and 3 MET was low intensity, 3 and 6 MET was moderate intensity and more than 6 MET was high intensity.

An HIG intervention session combined jogging and interactive games [Children Games (5 MET), teach physical education (6.5 MET), run at 5 to 7 miles per hour (mph) (8 at 11.5 MET)], which included muscle building (6 MET). Each session began with a warm-up and ended with muscle stretching. This program aimed to improve Leger’s VO2max score, muscle building, and overall physical fitness. For instance, sessions included a treasure hunt across a large perimeter jog/walk combination (6–6.5 MET), wheelbarrow racing, spider walking and reinforcement exercises through gym weights. For each participant, 75 to 80% of the theoretical maximum heart rate for their age was recommended with verification of their heart rate during the sessions.

Each LMIG intervention combined games that highlighted the sensation of (gentle and slow) movements (proprioception) with seated play [sitting with children plays (2.8 MET)], postural control [stretching, yoga (2.5 MET)] and interactive games [walking children’s games (MET 5), walk/run –playing with children, with moderate period (MET 4)]. Each session started with a warm-up and aimed not to focus on performance or exertion to the point of breathlessness. The sessions included, for example, a slow relay race with variations of movement, interactive games in teams (walking or running slowly whilst perpetuating equilibration), a treasure hunt in a small area, mime games, riddles and optical illusions.

In both interventions, the experimenters recorded the duration of all exercises and the participants’ feelings and heart rates. Positive remarks were regularly written in an individual file to motivate each participant. The exercises were explained, customized and set up by the therapists. Every effort has been made to create a non-competitive atmosphere (mockery was forbidden). At the end of each session, a similar gift was provided to each participant (juice and a pack of candies or gum). At the end of each week, an extra gift was given (a small toy, a game, stickers, etc.) to reward efforts and increase motivation.

### Primary Outcomes

The CDI is a 27-item self-report instrument (Kovacs and Beck, 1979) that provides a score based on the child’s statement describing how she or he felt during the two previous weeks. The average score in the general population is 9 points (Kovacs and Beck, 1979), and a score of 19 or more is considered pathological (Smucker et al., 1986). The CDI showed good internal reliability (Cronbach’s alpha = 0.86), sensitivity, specificity and a low level of bias (Stockings et al., 2015). The pooled estimate for internal reliability is considered as high. This scale is mainly used in research to assess mood and depression (ICD-10-CM F32.9) in a longitudinal follow-up.

The STAI (Spielberg et al., 1983) consists of two subscales with twenty items, the STAI, form Y-A, which assesses the recent state (i.e., what the participants felt during the week of testing), and the STAI, form Y-B, which assesses the long-term anxiety trait (i.e., what the participants felt during the past year). Each part (A or B) of the STAI provides a state score or an anxiety trait score ranging from 20 to 80. The pathological threshold is 39–40 (Knight et al., 1983). This test has a high degree of reliability (Cronbach’s alpha = 0.86) and validity. This is widely used to detect anxiety (ICD-10-CM F41.9).

In this study, the abbreviated BDI-13 (Beck et al., 1988), the Hospital Anxiety and Depression Scale (HADS) (Zigmond and Snaith, 1983), the Zung Self-Rating Depression Scale (SDS) (Zung, 1965), and the Self Perception profile for Children (Bouffard et al., 2002) were also used to determine their relevance and reliability in testing pre-adolescents.

The Beck Depression Inventory (BDI) (Beck et al., 1988) consists of 13 questions with multiple-choice answers evaluating depression severity. A Likert scale from 0 to 3 is assigned for each answer and the item scores are summed to give a total score ranging from 0 to 39. The higher the score is, the higher the severity of depression is. The BDI has a high internal consistency (alpha coefficients = 0.86 for non-clinical population).

The HADS is a reliable questionnaire (Zigmond and Snaith, 1983) that allows detecting anxiety and depressive disorders. It has 14 items rated from 0 to 3. There are 7 questions related to anxiety (subscale A) and 7 others to the depressive dimension (subscale D) providing 2 scores. Maximal scores for anxiety and for depression range from 0 to 21. A score of 0 to 7 for either subscale indicates an absence of symptoms while a score of 11 or more is probably pathological. The internal reliability is correct (Cronbach’s alpha = 0.79 to 0.86).

The Zung Self-Rating Depression Scale questionnaire (Zung, 1965) is used to assess depression severity. There are 20 items in the scale. Each question is scored on a Likert scale ranging from 1 (rarely) to 4 (nearly always). The sum of item scores is multiplied by 1.25 and divided by 100 to provide the depression score in percentage. Most people with depression have a score between 0.50 and 0.69 while a score of 0.70 and above indicates severe depression. According to the World Health Organization, the SDS score may give an indicative range for the severity of depression but is not sufficient to confirm the pathology. The internal consistency is correct (Cronbach’s alpha = 0.79).

To assess self-esteem, the Self Perception profile for Children (Harter, 1985) has been used in its French version, the “Questionnaire de perception de soi pour jeunes, QPSJ.” There are six categories including six questions, for a total of 36 questions. Each category refers to a competence (social, athletic, scholastic, physical, behavioral, and global self-worth). The internal consistency is acceptable to good (Cronbach’s alphas of the six categories are ranged 0.71 to 0.86).

### Secondary Outcomes

A body composition mass monitor (OMRON BF 511^®^), used in humans aged 6 to 80 years, was used to measure body mass, body fat, muscle percentage and skeletal muscle percentage. A medical device was also used to measure height.

The *Leger shuttle test* is a 20-meter multi-steps shuttle run test, providing an extrapolated assessment of maximal oxygen uptake (VO2max) (Leger et al., 1988). A meta-analysis showed that “*the 20 m shuttle test seems to be a useful alternative to estimate cardiorespiratory fitness*” and this test has a high validity in young people (Mayorga-Vega et al., 2015). We used POLAR FT2^®^ heart-rate monitors with a transmitter belt at the sternum level to record maximum heart rate, average heart rate, and session time.

Pre-assessment and post-assessment tests took place, respectively, the week before and the week after the intervention.

### Statistical Analysis

A sample size was defined from a previous study, whereby CDI II was used to measure variance in depression among adolescents receiving physical exercise as treatment (10). Accordingly, with a detected change of −9, a detected change in SD of 10.2, a desired power of 0.8 and a *p* = 0.05, 12 children per group were needed to obtain significant results.

Since some measures were based on Likert scores, a graphical tool (quantile–quantile plot) and a Kolmogorov Smirnov test were used to assess the distribution of normality. If the data were not normal, a logarithmic transformation was performed to obtain normal data (if it were possible). The General Linear Model (GLM), with two criteria, or the Mann–Whitney test was performed to determine the difference between the two groups and two tests sessions. Due to a prior assumption of greater changes in depression/anxiety scores for HIG, two dependent tests (*t*-test or Wilcoxon test) were used to determine the change in a variable (STAI, CDI, etc.) over time in each experimental group (HIG or LMIG). The effect size based on the partial eta squared (η^2^) was performed to calculate the number of participants required for future research. A Pearson correlation test was used to evaluate the potential correlation between the variables in the two groups. All statistical procedures were performed using the SPSS software. The data were anonymized for the statistical analysis so that the two groups could not be identified.

## Results

### Participation Analysis

**FIGURE 1:**
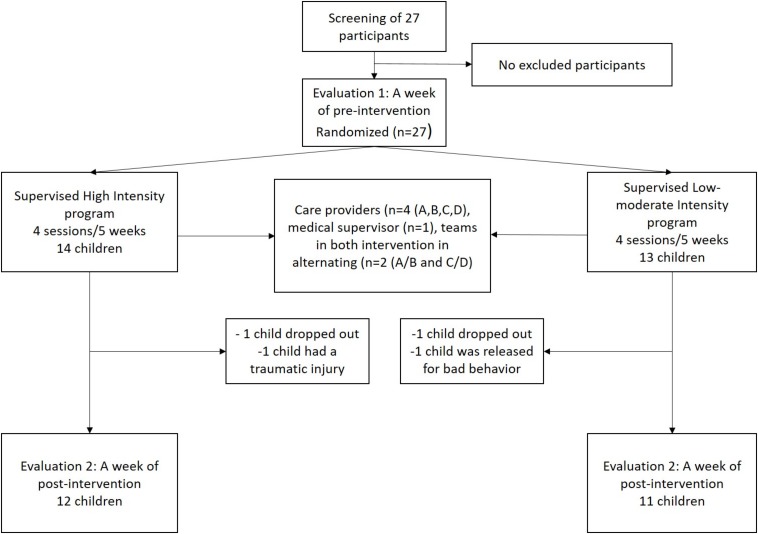
Flow chart of study design. Among the 27 pre-adolescents who were recruited and randomized (quantifiers: CDI, BMI, Léger shuttle test, age, sex, and school year), four participants were lost during the experimentation. The statistical analysis of T1 and T2 measures was restricted to the 23 participants involved in the whole study.

Among the recruited 27 pre-adolescents in pre-test, four participants, two in each group, were lost during the experimentation. The statistical analysis of pre- and post-test measures was restricted to the remaining 23 participants who were involved in the whole study (see Figure 1 for details). The mean age at assessment was 10.7 ± 0.7 with 6 girls and 6 boys in HIG and 10.6 ± 0.7 with 5 girls and 6 boys in LMIG (*p* = 0.690, Student *t*-test).

The mean attendance rate was 93.11% in HIG and 96.65% in LMIG (*p* = 0.290, Mann–Whitney test). The mean individual “tiredness score” (0–3, 0 = not tired at all; 1 = a little bit tired; 2 = moderately tired; 3 = exhaustively tired), averaged for all sessions, was slightly higher in HIG than LMIG (0.64 versus 0.43) without significant difference between groups (*p* = 0.600, Mann–Whitney test). The participants enjoyed the interventions, as reported in a mean “fun index” (0–3, 0 = very fun; 1 = quite fun; 2 = moderately boring; 3 = very boring), averaged for all sessions, which revealed no difference between groups (0.45 in HIG and 0.52 in LMIG, *p* = 0.710, Mann–Whitney test).

A HIG intervention session lasted an average of 43 min, combining 15 min of jogging and interactive games, 15 min of muscle building and 8 min of warm-up and stretching. Each LMIG intervention lasted also an average of 43 min, combining 16 min of seated play, postural control and very gentle movements, 23 min of games exerting the sensation of movement (proprioception), with gentle and slow movements, and a warm-up (2 min).

### Primary Outcome

Self-reported data from primary questionnaires are summarized in Table 1. The Likert score variables, with a non-normal distribution, were the CDI and the BDI-13. However, a transformation was made on the CDI and BDI scores with this formula “Ln(variable)” to obtain a normal distribution. Only CDI score variables became normal after this procedure.

**TABLE 1:** Mean scores in Self-reports for both groups over time.

		**High intensity group**		**Low-to-moderate intensity group**				
**Self-reports**	**Time**	**Mean (SEM)**	***p*-value (T2-T1)°**	**Mean (SEM)**	***P*-value (T2-T1)°**	Group × time effect^*^	Group effect^*^	Time effect^*^
STAI A	T1	29.25 (2.47)	0.856	30.55 (2.60)	0.628	0.690	0.868	0.912
	T2	29.83 (2.78)		29.36 (2.04)				
STAI B	T1	36.58 (2.75)	0.911	38.82 (2.20)	**0.004**	**0.050**	0.912	**0.030**
	T2	36.33 (2.30)		33.36 (2.83)				
CDI	T1	11.00 (2.75)	0.447	10.36 (2.83)	**0.006**	0.250	0.480	**0.007**
	T2	10.08 (2.98)		6.73 (1.88)				
SDS	T1	0.49 (0.03)	0.342	0.48 (0.04)	0.518	0.79	0.83	0.25
	T2	0.47 (0.03)		0.46 (0.03)				
BDI-13	T1	2.42 (1.12)	0.953	4.00 (1.24)	0.719	0.71	/	/
	T2	2.58 (1.08)		3.64 (1.68)				
HADS A	T1	6.75 (1.18)	0.749	6.27 (1.24)	0.939	0.785	0.854	0.876
	T2	6.42 (1.17)		6.36 (0.91)				
HADS D	T1	3.58 (0.86)	0. 874	4.36 (0.45)	0.242	0.360	0.733	0.273
	T2	3.50 (1.026)		4.45 (0.73)				
QPSJ A	T1	2.98 (0.17)	0.633	2.92 (0.18)	0.858	0.617	0.645	0.726
	T2	3.07 (0.20)		2.90 (0.19)				
QPSJ B	T1	2.75 (0.17)	0.805	2.98 (0.18)	0.518	0.967	0.419	0.676
	T2	2.80 (0.28)		3.03 (0.20)				
QPSJ C	T1	2.86 (0.11)	0.810	2.60 (0.20)	0.740	0.711	0.386	0.970
	T2	2.80 (0.24)		2.65 (0.13)				
QPSJ D	T1	3.02 (0.25)	0.722	2.92 (0.16)	0.918	0.781	0.798	0.925
	T2	2.99 (0.27)		2.93 (0.18)				
QPSJ E	T1	2.87 (0.13)	0.655	2.96 (0.16)	0.715	0.578	0.887	0.870
	T2	2.95 (0.19)		2.92 (0.18)				
QPSJ F	T1	2.95 (0.18)	0.719	2.96 (0.18)	0.161	0.522	0.779	0.273
	T2	3.00 (0.22)		3.13 (0.19)				

Bold values indicates *P*-value < 0.05; SEM, standard error of the mean; *p*-Value from dependent test; ^*^p-Value from independent *t*-test; Details of the denomination of self-reports in the manuscript.

It should be noted that, although participants were randomly assigned, STAI-B scores at baseline (T1) were slightly higher in LMIG than in HIG. However, the group difference was not significant (*p* = 0.52, unpaired *t*-test). A GLM performed on the STAI-B scores revealed a significant group × time interaction (*p* = 0.050) and a time effect (*p* = 0.030). With respect to the LMIG, we found that this program induced lower anxiety symptoms over time with a significant and reproducible effect in small samples [*F*(1,21) = 4.17, *p* = 0.050, η^2^ = 0.17]. The eta-squared (η^2^) measure from independent *t*-test ranges between 0 and 1. Following the Cohen guidelines (Cohen, 1988) for the eta-squared (small effect = 0.01, moderate effect = 0.06, and large effect = 0.14), the outcome of η^2^ = 0.17 showed a large effect size.

**FIGURE 2:**
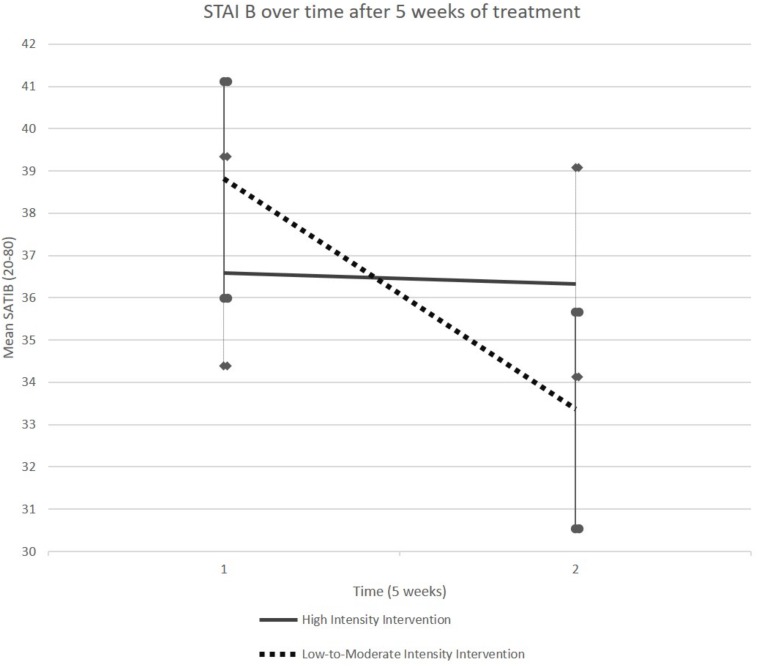
Evolution of STAI-B score. The STAI-B scores, on average, decreased between T1 and T2 in LMIG (*p* = 0.004) whereas these scores remained on average stable in HIG. The cut-off considered for pathological score was 39–40.

It was only in the LMIG that the STAI B scores, on average, decreased from 38.82 (SEM (Standard Error of Mean) 2.20) at T1 to 33.36 (SEM 2.83) at T2 (*p* = 0.004, Student paired *t*-test, see Figure 2 and Table 1). On average, the scores remained stable in HIG, evolving from 36.58 (SEM 2.75) to 36.33 (SEM 2.30).

Moreover, analysis of data from the children with “pathological” scores (39 and higher), with a GLM performed on their STAI-B scores, revealed no group × time interaction effect (*p* = 0.270). Among children with “pathological” scores, only those who attended LMIG sessions improved their STAI-B scores at post-test [36.17 ± (SEM 1.65)] relative to the pre-test [44.00 ± (SEM 2.89)] (*p* = 0.019, Student paired *t*-test).

The analysis of STAI-A scores did not reveal any group difference nor time-dependent changes (all *p*-Values > 0.05, see Table 1).

**FIGURE 3:**
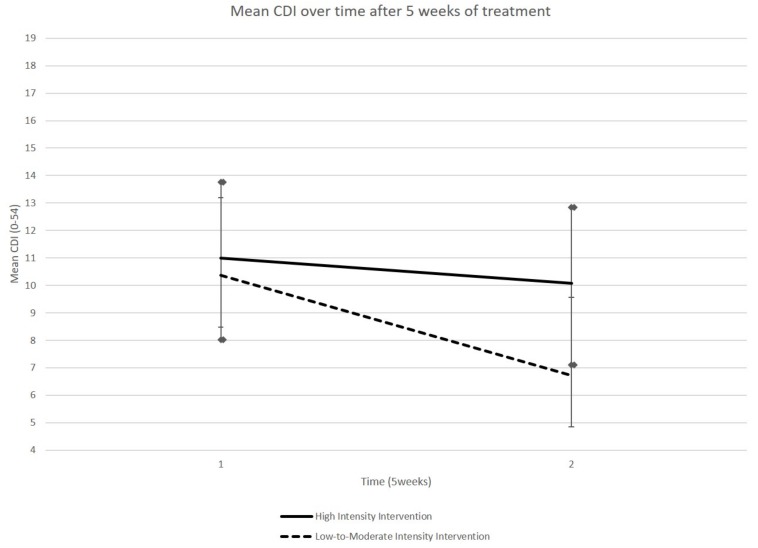
Evolution of CDI score. The CDI scores, on average, decreased between T1 and T2 in LMIG (*p* = 0.006) whereas these scores remained on average stable in HIG. The cut-off considered for pathological score was 19.

A GLM performed on the CDI scores did not reveal a group × time interaction (*p*-Value = 0.25) but a time effect (*p*-Value = 0.007). Concerning the LMIG, we observed that this program induced a moderate size effect, resulting from the eta squared, to lower depressive symptoms, although the results did not reach the level of significance [*F*(1,21) = 1.60, *p* = 0.25, η^2^ = 0.071]. It was only in the LMIG that a significant improvement in CDI scores was observed; the CDI scores decreased, on average, from 10.36 (SEM 2.83) at T1 to 6.73 (SEM 1.88) at T2 (*p* = 0.006, Student’s paired *t*-test, see Figure 3 and Table 1). The average scores remained stable in HIG, evolving from 11.00 (SEM 2.75) to 10.08 (SEM 2.98) (*p* > 0.05, Student *t*-test).

The GLM analysis of pathological CDI scores (19 and higher) was not considered since only four patients (2 in each group) had pathological scores.

The STAI A, the abbreviated BDI-13, the HADS, the SDS and the Self Perception profile for Children did not reveal any group difference for depression or for anxiety (all *p*-Value > 0.05).

### Secondary Outcome

These data are summarized in Table 2. The Likert score variables, with a non-normal distribution, were the BMI and the body fat. A transformation was made on these two variables scores with the formula “Ln(variable)” to obtain a normal distribution. However, the two variable scores remained non-normal after the procedure.

**TABLE 2:** Mean scores in physical tests for both groups over time.

		**High intensity group**		**Low-to-moderate intensity group**				
**Physical measures**	**Time**	**Mean (SEM)**	***p*-value (T2-T1)°**	**Mean (SEM)**	***P*-value (T2-T1)°**	Group × time effect^*^	Group effect^*^	Time effect^*^
Size (cm)	T1	143.88 (2.63)	**0.010**	139.72 (3.05)	**0.001**	0.36	0.30	**0.00**
	T2	144.71 (2.68)		140.27 (3.12)				
Body mass (kg)	T1	37.19 (3.26)	**0.003**	34.28 (1.74)	**0.004**	0.54	0.43	**0.00**
	T2	38.1 (3.21)		35.00 (1.74)				
Body mass index (Kg/m^2^)	T1	17.68 (0.95)	**0.040**	17.49 (0.54)	0.075	0.97	*/*	***/***
	T2	17.88 (0.91)		17.97 (0.54)				
Body fat (% of body mass)	T1	17.69 (2.11)	**0.003**	19.1 (1.74)	**0.033**	0.47	***/***	***/***
	T2	19.52 (2.02)		21.17 (1.76)				
Muscular mass (% of body mass)	T1	33.5 (0.79)	0.464	33.43 (1.13)	**0.033**	0.25	0.75	**0.03**
	T2	33.19 (0.66)		32.44 (1.15)				
Resting metabolic rate (cal)	T1	1238 (46.15)	0.069	1213.45 (32.22)	0.387	0.44	0.63	**0.05**
	T2	1247.25 (44.90)		1217.63 (30.84)				
VO2peak (ml/min/kg)	T1	42.21 (1.53)	**0.014**	42.45 (1.77)	0.740	0.174	0.60	0.06
	T2	44.88 (1.46)		42.89 (1.34)				

Bold values indicates *P*-Value < 0.05; SEM, standard error of the mean; *p*-Value from dependent test; ^*^p-Value from independent *t*-test; VO2peak, maximal oxygen consumption.

When a GLM was used to analyze physical measures, it did not reveal a group × time interaction (*p* > 0.05). The children’s weight and size increased similarly in each group at the end of the 5-week program. There was no difference between groups regarding weight, height, body mass index, muscle or body fat percentages, nor at T1, nor at T2 (all *p*-Values > 0.05).

**FIGURE 4:**
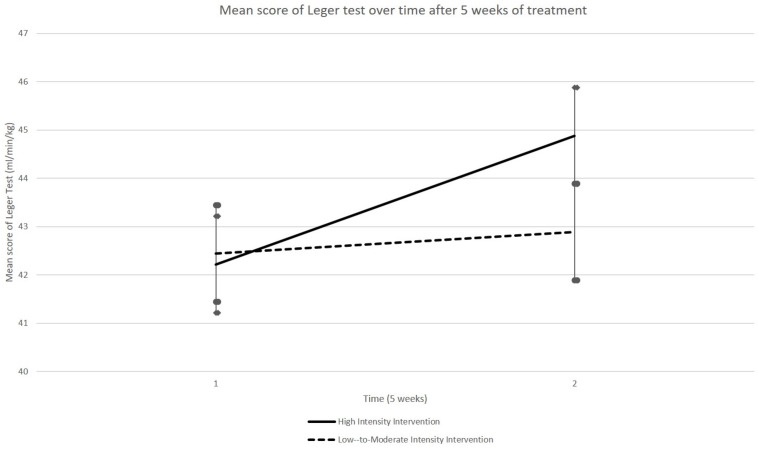
Evolution of VO2max indices. The VO2max indices as evaluated using the Léger shuttle test, on average, evolved better between T1 and T2 in HIG compared to LMIG. Although there was a trend for improvement of these indices in both groups, a significant improvement of VO2max indices (*p* = 0.014) was only observed in the HIG group. The errors bars represent standard error of the mean (SEM).

A GLM performed on the VO2max estimations, as evaluated using the Léger shuttle test, did not reveal any group × time interaction (*p* = 0.174). Although there was a trend for improvement in both groups (time effect: *p* = 0.060), it was only in HIG that a significant improvement of VO2max estimation was observed at T2 [44.88 (SEM 1.46) ml/min/kg] relative to T1 [42.21 (SEM 1.53) ml/min/kg] (*p* = 0.014, paired *t*-test, see Figure 4). The scores remained generally stable in LMIG, evolving from 42.45 (SEM 1.34) ml/min/kg at T1 to 42.89 ± (SEM 1.77) ml/min/kg at T2.

Additional Pearson correlation analyses were performed to test the relationship between the changes in CDI/STAI-B/Léger shuttle test over time (T2-T1). Only the variables that showed a significant time-related change in paired *t*-tests were analyzed according to the Pearson correlation. The pre-test scores were, first, subtracted from the post-test scores for each variable (CDI, STAI-B, and VO2max) and the obtained indexes of time-related change were further correlated with each other. This analysis did not reveal any correlation between variables.

## Discussion

This study sought to compare high-intensity and low- to moderate-intensity physical exercise programs, as per their impact on self-reported depression and/or anxiety scores among a non-clinical sample of 5th–6th grade pupils. The results of the participants from both groups were examined separately, and we found that CDI (self-report scale for depression) and STAI-B (self-report scale for trait anxiety) scores significantly improved, over time, in the LMIG. The HIG did not show this improvement, but there was an upward trend in improving the VO2max indices compared to the LMIG group.

### Main Results

Trials investigating the influence of PA on anxiety/depression symptoms in children and adolescents are few. They have yielded different results in terms of duration and intensity despite usually indicating that PA should have a beneficial effect on depressive symptoms (Korczak et al., 2017a). For instance, a study involving 43 adolescents with depression, where the intervention was a 6-week circuit-training made of 12 separate sessions done twice weekly at preferred intensity (Carter et al., 2015), saw significant beneficial effects on CDI 2 version scores. However, these improvements were only statistically significant at a 6 month follow up post-intervention and were not apparent immediately after its duration. When the sample size was measured from this previous study alongside the significant follow-up results, 12 children could be said to have markedly reduced levels of depressive symptoms because of the exercise therapy. The follow-up results were used to obtain the necessary sample size for our post-intervention because our program contained more sessions (20 sessions). Without lost participants, we would have been able to perform a significant change. Eventually, we performed a 5-week program, which is a short time period in comparison with another randomized clinical trial (Hughes et al., 2013). An intervention of 6 to 12 weeks was used and effective in alleviating depression in adolescents aged 12 to 18. Although we intended to do 50-min sessions, the average duration was 43 min approximately in each group. However, this duration was sufficient in demonstrating effective change, and average attendance was very good (92.21%) in both groups. A precise monitoring of the interventions, with continuous supervision on site, ensured that participants correctly performed the requested exercises with little deviation.

Thus, twenty sessions in 5 weeks seems adapted to decrease depression symptoms at post-intervention. According to a systematic review based on a meta-analysis mixing children and adolescents (Brown et al., 2013), PA could have a significant but small effect in the prevention and treatment of depression. A recent study in a large community-based sample of participants indicated that PA, objectively measured as moderate to vigorous, at 6 and 8 years of age lowered the average level of depressive symptoms 2 years later (Zahl et al., 2017). Our observations indicate that a high-intensity exercise intervention would not be the optimum to improve anxiety and depression symptoms in pre-adolescents, despite a better increase of physical condition. Even with positive coaching, high-intensity training can be restricting for some participants who feel unable to achieve their goals due to poor physical condition caused by a lack of prior exercise. In independent *t*-test in STAI-B, the eta squared value (η^2^ = 0.17) demonstrated a large effect size and can thus suggest that the children in our study benefited from the low-to-moderate program to reduce their anxiety symptoms compared to the high intensity program. Accordingly, combining low- to moderate-intensity exercise with interaction and play should better reduce the anxiety/depression symptoms. Additional studies are clearly needed to test this hypothesis in adolescents with a medical diagnosis of depression and/or anxiety.

### Limitations

A first limitation concerns our population who was highly heterogeneous with regards to depression/anxiety symptoms; it was not a clinical population, which also explains why somevariables did not have a normal distribution. The self-rated questionnaires used in this study are regarded as highly validand reliable (Stockings et al., 2015), although CDI and STAI cannot be considered substitutes for a medical diagnosis made by a psychiatrist (Rush et al., 2006). Thus, the results may not be inferred to patients since participants, even those who had scores above the cut-offs at pre-test, were not submitted to a neuropsychiatric interview. Secondly, with a small sample size (23 participants in the both groups), caution must be applied, as the findings for a reduction of anxiety in LMIG might not be for the reduction of anxiety score. Furthermore, the small sample size might be a contributing factor to the non-significance group × time effect in independent *t*-test for depression symptoms. It is therefore likely that more participants should be included in studies to evaluate the efficiency of the low-to-moderate intensity among pre-adolescents to reduce the psychological symptoms. Another potential problem is that we cannot exclude the possibility that participants of each group knew what their peers in the other group were doing. We attempted every effort to avoid mutual influence; the different questionnaires were used in random order to minimize outside influence. Then the inferred results were obtained from tests performed twice, once before and once after the 5-week period. This gives us a limited scope of data and knowledge, especially concerning any long-term effects of the intervention; a 6-month follow up examination would be required to rectify this problem but could not be achieved due to practicalities. In addition, when we tried to correlate changes in physical condition with changes in depression/anxiety scores there was no significant correlation, although poor physical condition may promote increased scores of depression due to the prevention effect of PA described in the literature (Hughes et al., 2013; Korczak et al., 2017a). It should be noted, however, that any correlation should not allow disentangling between the two hypotheses in terms of cause and effect (lack of PA favoring depression, depression favoring lack of PA).

## Ethics Statement

The ethical committee of the School of Medicine of the Université catholique de Louvain approved this study (No. CEHF-FORM-003/REV005–B403201627586).

## Author Contributions

AP contributed to the study design, was involved in the two arms of the trial, made the statistical analysis of data, and wrote the first draft of the manuscript. AM, LD, and GS were involved in the two arms of the trial and contributed to the statistical analysis. YB contributed to the study design and manuscript draft. ADV made the study design, verified the exercise and control arms, made all scoring, provided results on an anonymous basis, and wrote the manuscript. All authors had access to the study data that support the publication.

## Conflict of Interest Statement

The authors declare that the research was conducted in the absence of any commercial or financial relationships that could be construed as a potential conflict of interest.

## Abbreviations

CDI: Child Depression Inventory
HIG: high intensity group
LMIG: low-to-moderate intensity group
STAI: State-Trait Anxiety inventory.
